# Antibiotic resistance pattern and spa types of Staphylococcus aureus strains isolated from food business and hospital kitchen employees in Çanakkale, Turkey

**DOI:** 10.3906/sag-1712-207

**Published:** 2019-04-18

**Authors:** Nesrin ÇAKICI, Alper AKÇALI, Nükhet Nilüfer DEMİREL ZORBA

**Affiliations:** 1 Health Services Vocational School, Çanakkale Onsekiz Mart University, Çanakkale Turkey; 2 Department of Medical Microbiology, Faculty of Medicine, Çanakkale Onsekiz Mart University, Çanakkale Turkey; 3 Department of Food Engineering, Faculty of Engineering, Çanakkale Onsekiz Mart University, Çanakkale Turkey

**Keywords:** Food handler, food employees, *Staphylococcus aureus*, antibiotic resistance, *spa*typing

## Abstract

**Background/aim:**

The aim of this study was to determine antibiotic resistance profiles and *spa* types of *Staphylococcus aureus* strains isolated from food business employees in Çanakkale, Turkey.

**Materials and methods:**

*S.* *aureus* isolates were collected from hand and nasal swabs of 300 individuals working in 17 food businesses and 9 hospital kitchens. All *S. aureus* isolates obtained from each carrier were typed by staphylococcal protein A (*spa*) typing method.**Staphylococcal cassette chromosome *mec* (SCC*mec*) and multilocus sequence typing (MLST) of MRSA were performed by sequencing method.

**Results:**

Of the 300 individuals, 125 (41.6%) were found to be carriers of *S. aureus*, 215 isolates of which were obtained in total. Three (1.4%) of 215 isolates were identified as MRSA. Sixty *spa* types were identified among the 121 MSSA isolates, the most common being t084 (9%). A novel *spa* type was discerned and added to the Ridom SpaServer database as t14963. The MLST type of the MRSA strains identified as spa type t786 was ST88 and as spa type t223 was ST22. All MRSA were determined to be SCC*mec* type IVa.

**Conclusion:**

*spa* typing can be performed to screen for transmission of *S. aureus*. t786, ST88, and SCC*mec* IVa MRSA strains were identified for the first time in Turkey.

## 1. Introduction

*Staphylococcus aureus* is an important organism in public health, as a cause of nosocomial infections and food poisoning incidents. Owing to its potential resistance to multiple antibiotics and production of virulence factors, *S. aureus* represents an important source of both nosocomial and community-acquired infections (1,2).

Molecular typing has become increasingly important both for controlling the spread of *S. aureus *and determining the origins of epidemics associated with virulence factors (3). Staphylococcal protein A (*spa*) typing, which relies only on the assessment of repeats at the x region of *spa*, exhibits excellent discriminatory power and shares with multilocus sequence typing (MLST) the advantages of unambiguous typing results that can be compared between laboratories and over time. *spa* typing is also both easier and less costly to perform than MLST or PFGE (4,5). The x region exhibits high variability and may be useful for the rapid typing of MRSA, particularly in hospital environments. *spa* typing has been found to be a fast and practical method for identifying the causative organisms of epidemics, since it involves a single locus (5,6). A strong correlation has been demonstrated between the results of other techniques and the clonal groups obtained with *spa* typing. The importance of this method in epidemiological studies and the determination of clonal associations is gradually increasing (7,8).

Software such as Ridom StaphType enables the description of *spa* repeats and types, using consistent *spa* code terminology (6,9). Alternative free programs have also been developed to evaluate *spa *region sequencing data. The Java-based program DNAGear, developed on the NetBeans platform, is one example (5).

The aim of the present study was to determine the antibiotic resistance profiles and *spa* types of *S. aureus* strains isolated from catering employees in hospitals and food businesses (restaurants and catering industries) located in Çanakkale, Turkey, and to determine transmission events between hospitals and food businesses.

## 2. Materials and methods

### 2.1. Sample collection and strain isolation

Between April and December 2014, three samples were collected from each of 300 food business employees (chefs, sous-chefs, waiting staff, and kitchen cleaners) by swabbing the right and left hands (bare fingers, thumbs, and palms) and nasal mucosa. Individuals were a random sample of workers in their institutions. Of these, 228 were employed by food businesses (restaurants and catering industries), and 72 worked in hospitals in the city center and districts of Çanakkale. The swabs were used to inoculate 5 mL of sterile Brain Heart Infusion Broth (Oxoid). Inocula were then streaked onto Baird–Parker Agar medium (Merck) containing egg yolk emulsion. After incubation at 37 °C for 24 h, the purity of the resulting gray-black colonies surrounded by a 1–1.5 mm clear zone was verified before their use in further analyses. *S. aureus* isolates were identified morphologically by Gram staining and catalase and coagulase testing. The study was conducted using a protocol approved by the Local Ethics Committee of Clinical Research (Decision No. 050.99–214), and written informed consent was obtained from all participants.

### 2.2. Antimicrobial susceptibility testing

The disk diffusion method was performed, as described by the Clinical and Laboratory Standards Institute (CLSI), to test the susceptibility of *S. aureus* strains to methicillin (using 30 μg of cefoxitin) and 15 other antibiotics (amoxicillin/clavulanic acid, cefazolin, cefoperazone, cefotaxime, ceftazidime, cefaclor, imipenem, meropenem, gentamicin, erythromycin, tetracycline, ciprofloxacin, chloramphenicol, rifampin, and linezolid) (10).

### 2.3. DNA extraction

Several colonies obtained from a fresh culture were mixed thoroughly in tubes containing 50 µL of buffer solution from a stock comprising 925 µL of H2O, 25 µL of 10% SDS (Sigma), and 50 µL of 2 M NaOH (Merck). The tubes were then incubated at 100 °C for 10 min in a****TDB-120 dry heating block (Biosan) to lyse cells. Tris-EDTA buffer (50 µL; Sigma) was subsequently added and the tubes were centrifuged at 13,000 rpm for 10 min. The supernatant (40 µL) was collected from each centrifuged sample and added to 150 µL of Tris-EDTA buffer.

### 2.4. Detection of mecA genes

A ready-to-use mixture (2X ExPrime Taq Premix; Genet Bio) was employed for PCR amplification. Reactions were performed using *mecA*-F (5′-CCTAGTAAAGCTCCGGAA-3′) and *mecA*-R (5′-CTAGTCCATTCGGTCCA-3′) primers (Sentromer), which amplify a product of 314 bp (11).

### 2.5. spa typing

The polymorphic x region was amplified as previously described using primers *spa *1113F (5′-TAAAGACGATCCTTCGGTGAGC-3′) and 1514R (5′-CAGCAGTAGTGCCGTTTGCTT-3′) (Figure). Sequence analysis was performed by the Sanger sequencing method, and forward and reverse sequences were obtained for each sample. Isolate *spa* types were determined with the program DNAGear, which retrieves the updated *spa* type and repeat data automatically from the Ridom SpaServer database (5). The discriminatory power of the *spa* typing method with which the present data were obtained was calculated using an online application designed according to the formula proposed by Hunter (http://insilico.ehu.es/mini_tools/discriminatory_power) (12).

**Figure:**
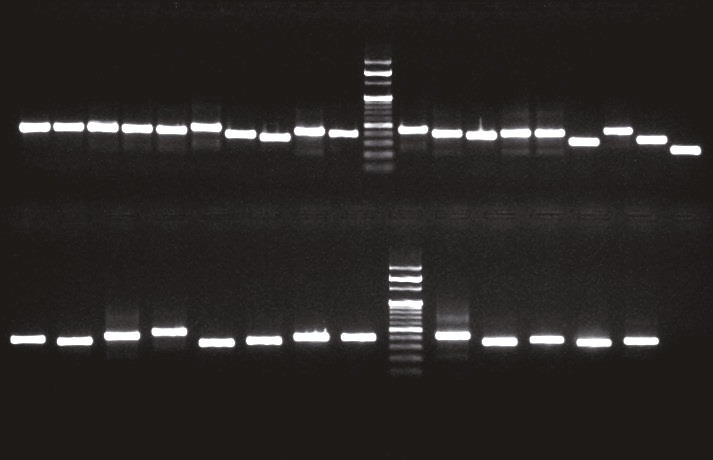
Region X of the spa genes of S. aureus isolates. 1–31: Isolate numbers. M: 100-bp DNA ladder. PC: Positive control (S. aureus, ATCC 25923). NC: Negative control (sterile water).

### 2.6. SCCmec and MLST typing

SCC*mec* types were determined by PCR analysis of the cassette chromosome recombinase (*ccr*) and *mec *gene complexes, as previously described (13,14). MLST analysis was performed by amplifying 7 gene regions for each clone as described online (http://saureus.mlst.net/misc/info.asp) (15).

### 2.7. Statistical analysis

Descriptive statistical analysis of the study data was performed with SPSS 19.0 (IBM Corp.).

## 3. Results

### 3.1. Strain isolation and antimicrobial susceptibility

*S. aureus* bacteria were isolated from both right and left hands and the nasal mucosa of 29 employees (n = 87). Additionally, these strains were isolated from the nasal mucosa and the right hands of 9 people (n = 18), the nasal mucosa and the left hands of 11 people (n = 22), the right and left hands of 12 people (n = 24), the right hands of 15 people (n = 15), the left hands of 8 people (n = 8), and the nasal mucosa of 41 (n = 41) people. Of the 300 participants in this study, 125 (41.6%) were found to be carriers of *S. aureus*, 215 isolates of which were obtained in total. Of the 215 *S. aureus* isolates, 90 were isolated from the nasal mucosa, 65 were from right hands, and 60 were from left hands.

Antibiotic resistance among these isolates was as follows: 19 (8.8%) were resistant to erythromycin, 15 (6.9%) to tetracycline, and 3 (1.4%) to cefoxitin. The cefoxitin-resistant isolates were obtained from nasal mucosa. These isolates were found to be susceptible to other antibiotics tested.

### 3.2. Determination of methicillin resistance by PCR

Three MRSA strains were isolated from the nasal mucosa of 3 food employees. Two individuals from whom MRSA strains were isolated were employees of food businesses and one worked in a hospital kitchen. The****MRSA carrier rate among the participants of this study was 1%.

### 3.3. spa typing of S. aureus isolates

An isolate was taken from each carrier for *spa* typing. If one individual carried *S. aureus* in more than one body region, the isolates isolated from the nasal mucosa, right hand, and left hand were selected for genotyping, respectively. Therefore, only one isolate of every person was taken for genotyping studies. Of the 125 *S. aureus* strains subjected to* spa* typing, 90 (72%) were isolated from nasal mucosa, 27 (21.6%) from right hands, and 8 (6.4%) left hands. Thirty-two (25.6%) isolates were obtained from hospital kitchen workers and 93 (74.4%) from food business employees. The *spa* gene region of one isolate could not be amplified with the primers used in MSSA isolates.

Sixty *spa* types were identified among the 121 MSSA isolates, of which 11 (9.0%) were t084, 9 (7.4%) t008, 8 (6.6%) t005, 6 (4.9%) each of t012 and t267, 5 (4.1%) t091, 4 (3.3%) each of t010 and t571, and 3 (2.5%) each of t223, t189, t310, t346, and t774. Furthermore, types t065, t158, t160, t304, t537, and t3266 were detected in two isolates each*.* Finally, 41 *spa* types were represented by single isolate. The distribution of *spa* types according to food businesses and hospitals is given in Table 1. The distribution of 121 MSSA isolates between food businesses (restaurants and catering industries) and hospitals according to *spa* types is shown in Tables 2 and 3. The *spa* types of the MRSA (n = 3) isolates obtained from food business employees were t786 and t223, and that taken from the hospital kitchen worker was of type t223. A total of 5 isolates were identified as *spa* type t223. Two of these were MRSA and 3 were MSSA.

**Table 1:** Distribution of spa types according to food businesses and hospitals.

spatype	Food businesses (n)	Hospitals(n)	Total MSSA, n (%)
t002	1	0	1 (0.8)
t004	1	0	1 (0.8)
t005	7	1	8 (6.6)
t008	8	1	9 (7.4)
t010	0	4	4 (3.3)
t012	5	1	6 (4.9)
t018	1	0	1 (0.8)
t026	0	1	1 (0.8)
t065	2	0	2 (1.6)
t084	9	2	11 (9.0)
t091	5	0	5 (4.1)
t136	0	1	1 (0.8)
t153	0	1	1 (0.8)
t156	1	0	1 (0.8)
t158	2	0	2 (1.6)
t160	2	0	2 (1.6)
t186	0	1	1 (0.8)
t189	3	0	3 (2.5)
t197	0	1	1 (0.8)
t223	0	3	3 (2.5)
t267	5	1	6 (4.9)
t284	0	1	1 (0.8)
t295	0	1	1 (0.8)
t304	2	0	2 (1.6)
t310	3	0	3 (2.5)
t335	1	0	1 (0.8)
t338	1	0	1 (0.8)
t346	3	0	3 (2.5)
t359	1	0	1 (0.8)
t537	2	0	2 (1.6)

**Table 2:** Distribution of MSSA isolates among restaurants and catering industries.

Sites	spa types (n)	Most frequentspa type (%)	MSSA (n)
C1	t158 (1)	-	1
C2	t091 (1), t537 (1), t1326 (1)	-	3
C3	t091 (2), t3266 (1)	t091 (67)	3
C4	t012 (1), t091 (1)	-	2
C5	t084 (3), t2243 (1), t3266 (1)	t084 (60)	5
C6	t005 (2), t008 (1), t002 (3), t160 (2), t2278 (1)	t002 (33)	9
C7	t004 (1), t065 (1), t189 (2), t310 (1), t537 (1), t693 (1), t774 (3), t2816 (1)	t774 (27)	11
C8	t008 (2), t084 (1), t267 (1), t843 (1), t9756 (1), t1097 (1), t12730 (1)	t008 (25)	8
C9	t005 (1), t018 (1), t084 (2), t156 (1), t723 (1), t1313 (1), t3906 (1), t4188 (1)	t084 (22)	9
C10	t005 (1), t008 (4), t012 (1), t304(1), t5480 (1)	t008 (50)	8
C11	t6367 (1), t10405 (1)	-	2
C12	t005 (1), t065 (1), t084 (1), t158 (1), t335 (1), t660 (1), t1236 (1), t6811 (1)	-	8
C13	t084 (1), t267 (2), t548 (1), t569 (1)	t267 (40)	5
C14	t008 (1), t267 (2), t304 (1), t310 (1), t338 (1), t359 (1)	t267 (28)	7
C15	t002 (1), t091 (1)	-	2
C16	t005 (1)	-	1
C17	t005 (1), t084 (1), t189 (1), t310 (1), t346 (3)	t346 (43)	7

C1, 2, 3, 4, 5, 6, 7, 10, 11, 12, 13, 14, 15, 16, 17: restaurants; C8 and C9: catering industries

**Table 3:** Distribution of MSSA isolates among hospitals.

Hospitals	spa types (n)	Most frequentspa type (%)	MSSA (n)
H1	t630 (1)	-	1
H2	t284 (1), t295 (1)	-	2
H3	t153 (1)	-	1
H4	t012 (1), t026 (1), t084 (1), t136 (1), t197 (1), t1439 (1), t14963 (1)	-	7
H5	t008 (1), t084 (1), t267 (1), t11108 (1)	-	4
H6	t005 (1), t186 (1), t571 (4), t1523 (1)	t571 (57)	7
H7	t865 (1)	-	1
H8	t223 (3)	t223 (100)	3
H9	t010 (4)	t010 (100)	4

H1–9: hospitals.

A novel, short repetitive region (04-44-33-31-12-16-12-33-34) determined by DNA sequence analysis to be present in isolate no. 101 has not previously been reported. This was added to the Ridom SpaServer database as “t14963” (http://spa.ridom.de/spatypes.shtml). The discriminatory power of the spa typing method in this study was calculated as 0.9736.

### 3.4. SCCmec and MLST typing of MRSA isolates

SCCmec analysis identified the 3 MRSA isolates as type IVa, suggesting that these strains were community-acquired MRSA (CA-MRSA). The MLST type of the MRSA strains identified as spa type t786 was ST88 and as spa type t223 was ST22 (Table 4).

**Table 4:** Genotypes and distribution of MRSA isolates.

Sites	spa types	SCCmec	MLST	Total MRSA (n)
C5	t786	IVa	ST88	1
C8	t223	IVa	ST22	1
H7	t223	IVa	ST22	1

C5: restaurant; C8: catering industry; H7: hospital.

## 4. Discussion

Recent studies have revealed that methicillin resistance is gradually increasing among CA-MRSA. A previous examination of 526 *S. aureus* isolates obtained from individuals employed in food businesses revealed 28 (5.3%) to be MRSA in a study performed in Antalya, Turkey, in 2013 (16). A similar investigation identified methicillin resistance in 17.1% of 169 *S. aureus* isolates recovered from nasal swabs of food employees in Bursa, Turkey, in 2010 (17). Another study of the carriage of this bacterium by individuals working in comparable roles reported that 0.4% of 73 isolates were MRSA in Alanya, Turkey (18). The prevalence of *S. aureus* methicillin resistance in our study was 1.4%, and 1% of the food employees tested carried MRSA. These values are lower than those reported by the majority of other investigations.

In the largest epidemiological survey ever done in Turkey to date, 397 MRSA strains collected from 12hospitals in 11 cities in different geographical regions of Turkey between 2006 and 2008 and 91 MRSA strains collected from 4 hospitals in 2011 were investigated in terms of *spa* types and clonalities according to MLST and PFGE methods. This study showed the presence of a hospital Turkish clone TR09 (ST239-SCC*mec*III-t030) and a community clone TR10 (ST737-SCC*mec*IV-t005) largely disseminated in Turkey. The most common MRSA clone in Turkey was found to be ST239 (91.4%)-SCC*mec* type III (91.4%), t030 (85.1%) (15). In a previous report, 54 MRSA strains obtained from 8 university hospitals, geographically distributed over the 6 main regions of Turkey, were shown to be of 4 *spa* types (t030, t459, t1459, and t189). t030 predominated, accounting for 89% of the isolates. All of them were ST239, SCC*mec* III (19). It was determined that all of the 48 MRSA isolates identified in the Eastern Black Sea Region except for 2 *spa* types were t030. The MLST types of strains representing the 3 different *spa* types were ST239 (3). In another study conducted on 9 MRSA strains isolated from blood cultures in İzmir, Turkey, it was shown that the dominant strain was t030 *spa* and it was a member of the clone ST239-IIIA (20). The most common *spa* and MLST types of 12 MRSA strains isolated from mastitic cow milk and nasal swabs of related agricultural workers in the Aydın region were t030, ST239. Ten of them were identified as SCC*mec*-III (21). In the latest work reported from Turkey, clinical MRSA isolates were collected from 6 university hospitals. Of the 270 MRSA isolates, 91% were found as SCC*mec* III and 81.1% as t030. The majority of MRSA isolates reported in Turkey belonged to ST239, t30, SCC*mec* III (22). We identified 2 of 3 MRSA strains as t223, ST22, SCC*mec* IVa and one as t786, ST88, SCC*mec* IVa. *spa* type t223, which was previously determined in Aydın, has also been determined in our work (15). ST22 was detected in the genotyping study of 102 MRSA strains at a university hospital in İstanbul (23).

*spa* type t223 MRSA isolates have been reported in many studies. In Jordan, 56 MRSA strains obtained from nasal swabs were identified as *spa* type t223 (39%). The majority of MRSA isolates harbored SCC*mec* IVa (75%) (24). *spa* type t223 was found to be the second most common type in Jordanian adults (25). The ST22-MRSA-IVa clone (*spa* t223) was dominant in the Gaza Strip and accounted for 64% of the MRSA in healthy children and their parents (26). In the Middle East, a study was conducted to investigate the molecular epidemiology of *S. aureus* outpatients attending primary healthcare centers in Egypt and Saudi Arabia. Only 2 *spa* types, t008 and t223, coexisted in both countries (27). In the Russian Federation, 9 of 13 CA-MRSA isolates belonged to ST22 (*spa* type t223, SCC*mec* type IVa) and were similar to the EMRSA-15/Middle Eastern variant (Gaza strain) (28). In Italy, 8 of 10 MRSA isolates obtained from nasal swabs of healthy children were ST22-MRSA-IVa, *spa* t223 (29). t223, one of the 10 most frequent *spa* types, originated from Sweden, Scandinavia, the rest of Europe, the Middle East, and Africa (30). *spa* type t786 MRSA has also been reported in a number of countries. A total of 1462 inpatients and healthcare workers were screened for MRSA nasal carriage in Sao Tome and Principe and Angola. The majority of the isolates (51.7%) belonged to *spa* types t186/t325/t786/t1814/t1951, ST88, and SCC*mec* type IVa, and such isolates were identified in both African countries (31). t786 (2) was reported in 20 MRSA isolates obtained from nasal swabs of outpatients attending primary healthcare centers in Egypt (27). In addition, t786 was detected in 381 MRSA strains obtained from patients admitted to 36 German hospitals (32). *spa* type t786 was also found in the Netherlands and Switzerland (33,34). In the majority of reported studies, it was found that the isolates of t786 were ST88, SCC*mec* type IVa. This strain type (t786, ST88) that we found has not been reported previously in Turkey.

In the current work, the same *spa* type (t223) was identified in the MRSA strains obtained from a food business employee and a hospital worker. MRSA may spread through a community via food business workers, and dissemination of a strain may occur from the wider community to a hospital, as well as in the opposite direction. Moreover, there is no relation between these MRSA carriers identified with the same *spa* type (t223) and they may have been colonized by different sources.

Over a time span of 6 months (May to October 2006), a total of 1490 staphylococcal isolates were sent to the National Reference Center in Germany for staphylococci for further characterization and typing. Among the 283 MSSA isolations, the most common *spa* types were t008 (14.5%) and t091 (4.9%) (7). In Hong Kong, food handlers from 6 catering establishments were nasally sampled for *S. aureus.* Of 434 food handlers, 99 (22.8%) were colonized with *S. aureus.* Forty-one *spa* types were determined within 94 MSSA isolates.* spa* typing revealed 18% of isolates as t189, 8.5% as t127, and 7.4% as t091 (35). A randomly selected, healthy Norwegian community population was studied for nasal carriage of *S. aureus* to describe frequency and molecular diversity. No MRSA isolates were found. The most frequent *spa* type t084 was detected in 7.4% (7/95) of the individuals. In our study, the most common *spa* type was t084 (36). In our study, the most common *spa* types of 121 MSSA isolates were t084 (9%), t008 (7.4%), and t005 (6.6%).

All MSSA isolates (n = 3) obtained from 3 individuals working in one of the district hospitals (H8) were of the same *spa* type (t223). Moreover, all MSSA isolates (n = 4) obtained from 4 individuals working in one of the hospitals in the province center (H9) were of the same *spa* type (t010). Here, MSSA isolates obtained from 2 hospitals were of the same *spa* type (t223 at H8 and t010 at H9), which was considered significant. This shows that the same clone may have spread to other people through a single person. Furthermore, 4 of the 7 isolates obtained from hospital H6 also shared the same *spa* type (t571). Interestingly, *spa* type t571, which we identified in 4 MSSA isolates, has been frequently found in many parts of the world among ST398 MSSA isolates, including isolates causing severe human infections. The ST398 genetic background is often associated with livestock-associated MRSA (37).

Here, 60 different *spa *types were detected among the MSSA isolates circulating in Çanakkale, Turkey. This draws attention to the fact that *spa* typing is also of use for MSSA strains. *spa* typing of MSSA isolates may contribute to identifying the source of any future local epidemics.

In conclusion, we have shown that rates of antibiotic resistance among *S. aureus* isolates obtained from food business employees and the carriage of MRSA by such individuals are low. This study contributed to the epidemiological data because of the detection t786, ST88, and SCC*mec* IVa MRSA strains, which have not been previously reported in Turkey.

**Acknowledgments**

This study was supported by the Scientific and Technological Research Council of Turkey (TÜBİTAK, SBAG Project No. 113S562). SCC*mec *and MLST**typing was performed by Prof Dr Bülent Bozdoğan’s team at the REDPROM Center of Adnan Menderes University in Aydın, Turkey. We would like to thank Dr John WA Rossen (University of Groningen, the Netherlands) for his help in determining the novel *spa *type.

## References

[ref0] (2002). Molecular genetics of methicillin-resistant Staphylococcus aureus. Int J Med Microbiol.

[ref1] (2012). Investigation of various virulence factors among the hospital and community-acquired Staphylococcus aureus isolates by Real-Time PCR. Microbiol Bul.

[ref2] (2014). Infectivity-resistotype-genotype clustering of methicillin-resistant Staphylococcus aureus strains in the central Blacksea Region of Turkey. Microbiol Bul.

[ref3] (2016). spa typing and multilocus sequence typing show comparable performance in a macroepidemiologic study of Staphylococcus aureus in the United States. Microb. Drug Resist.

[ref4] (2012). DNAGear—a free software for spa type identification in Staphylococcus aureus. BMC Research Notes.

[ref5] (2003). Typing of methicillin-resistant Staphylococcus aureus in a university hospital setting by using novel software for spa repeat determination and database management. J Clin Microbiol.

[ref6] (2008). spa typing of Staphylococcus aureus as a frontline tool in epidemiological typing. J Clin Microbiol.

[ref7] (2013). Overview of molecular typing methods for outbreak detection and epidemiological surveillance. Euro Surveillance.

[ref8] (2009). spa typing for epidemiological surveillance of Staphylococcus aureus. Methods Mol Biol.

[ref9] (2012). Clinical and Laboratory Standards Institute (CLSI). Performance Standards for Antimicrobial Disc Susceptibility Testing; Twenty-Second Informational Supplement.

[ref10] (2003). Multiplex PCR for the detection of genes encoding aminoglycoside modifying enzymes and methicillin resistance among staphylococcus species. J Korean Med Sci.

[ref11] (1988). Numerical index of the discriminatory ability of typing systems: an application of Simpson’s index of diversity. J Clin Microbiol.

[ref12] (2002). Multiplex PCR strategy for rapid identification of structural types and variants of the mec element in methicillin-resistant Staphylococcus aureus. Antimicrob Agents Chemother.

[ref13] (2005). Novel multiplex PCR assay for characterization and concomitant subtyping of staphylococcal cassette chromosome mec types I to V in methicillin-resistant Staphylococcus aureus. J Clin Microbiol.

[ref14] (2013). t030 is the most common spa type among methicillin-resistant Staphylococcus aureus strains isolated from Turkish hospitals. Microbiol Bul.

[ref15] (2013). Investigation of nasal Staphylococcus aureus carriage and methicillin resistance rates with three different methods in food handlers working in Antalya. Turkish Bulletin of Hygiene and Experimental Biology.

[ref16] (2010). Asymptomatic carriage of bacteria in food workers in Nilüfer District. Turk J Med Sci.

[ref17] (2011). Nasal and pharyngeal carriage of Staphylococcus aureus among hotel staff and risk assessment. Klimik Dergisi.

[ref18] (2009). MRSA genotypes in Turkey: persistence over 10 years of a single clone of ST239. J Infect.

[ref19] (2010). Hastanesi’ndeki dominant metisiline dirençli Staphylococcus aureus suşunun moleküler yöntemlerle tiplendirilmesi. ANKEM.

[ref20] (2013). Molecular typing of methicillin-resistant Staphylococcus aureus strains isolated from cows and farm workers. Journal of the Faculty of Veterinary Medicine of Kafkas University.

[ref21] (2016). Epidemiological and molecular characteristics of methicillin-resistant Staphylococcus aureus in Turkey: a multicenter study. J Global Antimicrob Resist.

[ref22] (2013). The high diversity of MRSA clones detected in a university hospital in Istanbul. Int J Med Sci.

[ref23] (2015). Molecular epidemiology of nasal isolates of methicillin-resistant Staphylococcus aureus from Jordan. Journal of Infection and Public Health.

[ref24] (2013). The epidemiology and molecular characterization of methicillin-resistant staphylococci sampled from a healthy Jordanian population. Epidemiol Infect.

[ref25] (2012). A typical hospital-acquired methicillin-resistant Staphylococcus aureus clone is widespread in the community in the Gaza Strip. PLoS One.

[ref26] (2015). Staphylococcus aureus nasal carriage among outpatients attending primary healthcare centers: a comparative study of two cities in Saudi Arabia and Egypt. Branz J Infect Dis.

[ref27] (2017). Molecular epidemiology and antibiotic resistance of methicillin-resistant Staphylococcus aureus circulating in the Russian Federation. Infect Genet Evol.

[ref28] (2014). -positive ST22 MRSA-IVa in healthy Italian preschool children. Infection.

[ref29] (2014). Epidemiology of MRSA in southern Sweden: strong relation to foreign country of origin, healthcare abroad and foreign travel. Eur J Clin Microbiol Infect Dis.

[ref30] (2015). Frequent occurrence of oxacillin-susceptible mecA-positive Staphylococcus aureus (OS-MRSA) strains in two African countries. J Antimicrob Chemother.

[ref31] (2009). Cross-border comparison of the admission prevalence and clonal structure of methicillin-resistant Staphylococcus aureus. J Hosp Infect.

[ref32] (2012). Prevalence of community-associated methicillin-resistant Staphylococcus aureus and Panton-Valentine leucocidin-positive S. aureus in general practice patients with skin and soft tissue infections in the northern and southern regions of the Netherlands. Eur J Clin Microbiol Infect Dis.

[ref33] (2013). Molecular epidemiology of methicillin-resistant Staphylococcus aureus in Switzerland: sampling only invasive isolates does not allow a representative description of the local diversity of clones. Clin Microbiol Infect.

[ref34] (2014). Occupational exposure to raw meat: a newly-recognized risk factor for Staphylococcus aureus nasal colonization amongst food handlers. International Journal Hygiene Environmental Health.

[ref35] (2011). Nasal carriage of Staphylococcus aureus: frequency and molecular diversity in a randomly sampled Norwegian community population. APMIS.

